# Genetic diversity, virulence factors and drug resistance of *Pantoea* strains isolated from samples of fresh fruits, vegetables and soil

**DOI:** 10.2478/jvetres-2025-0047

**Published:** 2025-09-13

**Authors:** Ewelina Farian, Katarzyna Kowalczyk, Teresa Kłapeć, Jacek Sroka, Piotr Skowron, Grzegorz Siebielec, Tamara Jadczyszyn, Jolanta Małgorzata Zdybel, Tomasz Cencek, Angelina Wójcik-Fatla

**Affiliations:** 1 Department of Health Biohazards and Parasitology, Institute of Rural Health, 20-090 Lublin, Poland; 2 Department of Parasitology and Invasive Diseases, Bee Diseases and Aquatic Animal Diseases, National Veterinary Research Institute, 24-100 Puławy, Poland; 3 Department of Plant Nutrition and Fertilization, Department of Soil Science Erosion and Land Protection, Institute of Soil Science and Plant Cultivation State Research Institute, 24-100 Puławy, Poland; 4 Department of Soil Science Erosion and Land Protection, Institute of Soil Science and Plant Cultivation State Research Institute, 24-100 Puławy, Poland

**Keywords:** ecological niche bacteria, facultative pathogen, food, *Pantoea*, plant pathogen

## Abstract

**Introduction:**

*Pantoea* is a genus of Gram-negative bacteria from the *Erwiniaceae* family. These bacteria are opportunistic human pathogens which are widely distributed in plants and soil. This study aimed to reveal the genetic diversity of *Pantoea* isolates from food and soil, characterise them biochemically and evaluate their drug resistance.

**Material and Methods:**

Thirty *Pantoea* strains were isolated from fresh fruit (n = 2), fresh and minimally processed vegetables (n = 12) and soil samples (n = 16). The genomic DNA was isolated from cultures on nutrient agar, and species were identified by amplification of 16S ribosomal RNA and housekeeping gene fragments and confirmed by sequencing. Virulence gene presence was determined by amplification of the *hcp* (haemolysin-coregulated protein), *vgrG* (glycine-valine repeat sequence G), *acrA* (anti–clustered regularly interspaced short palindromic repeat protein A) and *acrB* genes. Isolate drug resistance was tested using the disc-diffusion and gradient strip methods. The presence of Ambler class C (AmpC) β-lactamase (βL) and extended-spectrum (ES) βL resistance genes was tested for.

**Results:**

Five species were identified: *P. agglomerans* (n = 24), *P. ananatis* (n = 1), *P. eucalypti* (n = 1), *P. conspicua* (n = 1) and *P. vagans* (n = 2). The *hcp* and *vrgG* virulence genes were detected in 7 and 1 strain, respectively. All strains showed high resistance to cephazolin and cephuroxime, and more than half did so to ampicillin. The production of AmpC βL and ESβL was confirmed in 22 and 25 strains, respectively. Three strains of the *Pantoea* bacteria, including *P. ananatis* from leeks and *P. agglomerans* from arugula and soil, showed resistance to three or more antimicrobial classes.

**Conclusion:**

*Pantoea* spp., including multidrug-resistant strains, in fresh foods pose a potential risk of infection to consumers.

## Introduction

The *Pantoea* genus holds species which were formerly classified to the *Erwinia* or *Enterobacter* genus and comprises non-enveloped, non-spore-forming facultative anaerobic Gram-negative bacteria of the *Erwiniaceae* family. These bacteria are widespread in the natural environment, and their presence has been confirmed in various ecological niches, including soil and water (2). The *Pantoea* genus includes more than 20 species, mainly identified as epiphytes, endophytes or plant pathogens (16). However, these pathogens are also found in clinical samples, including blood, urine, pus and sputum (7).

The high phylogenetic similarity of individual species of the genus *Pantoea* and close kinship with other Gram-negative bacteria significantly hinder the identification of microorganisms and the determination of differences in the pathogenicity of environmental and clinical isolates. Phylogenetic analysis and knowledge of the source from which a strain was isolated are not sufficient for differentiating environmental from clinical isolates for their respective virulence. Strains isolated from the environment may have a similar pathogenic potential for humans as strains isolated in clinical conditions (34, 35, 46).

*Pantoea agglomerans* is the most widely studied species in the genus and is commonly found in both environmental and clinical settings. According to the classification of harmful biological agents in Poland set out in a Health Ministry regulation, the species *P. agglomerans* falls in risk group 2. The regulation indicates that this species is capable of producing toxins and causing an allergic reaction (21). *Pantoea agglomerans* primarily causes pneumonia and wound and urinary tract infections (7). This species is often recognised as the cause of soft tissue or bone and joint infections that result from skin trauma caused by wounding with plant material (7, 36, 46). It is also often referred to as the causative agent of occupational respiratory diseases among workers exposed to plant dust inhalation while processing wood, cereals or herbs (30). In addition, *P. agglomerans* has also been associated with sepsis, peritonitis, bacteraemia, meningitis, brain abscesses and bilateral endogenous endophthalmitis (20, 42, 45, 46). Other *Pantoea* species commonly found in the environment are also of pathogenic and clinical importance, including *P. dispersa, P. ananatis, P. septica, P. conspicua, P. brenneri, P. eucrina, P. gaviniae* and *P. calida*. The large number of these *Pantoea* species which also can be isolated from the clinical environment indicates that the genus *Pantoea* is a common opportunistic pathogen (32).

Bacteria of the *Pantoea* genus have several features, some genetic, that facilitate their colony formation and survival in various food products or soil and make then infectious to humans (41). One of the main threats to public health worldwide is antimicrobial resistance, especially among members of the *Enterobacterales* order, including the *Erwiniaceae* family. This threat prompts careful analysis of these microorganisms, and of *Pantoea* species no less than of others (7). According to the research conducted so far, clinical and environmental strains of the *Pantoea* genus are closely related, showing similarity in the occurrence of virulence genes and, therefore, in their virulence potential (16, 34).

This study aims to enhance understanding of the pathogenicity of *Pantoea* bacteria isolated from fresh food and soil. This is based on their biochemical characteristics, genetic identification, and drug resistance analysis in these opportunistic human pathogens.

## Material and Methods

### Sample collection

Unprocessed fruit (strawberries and raspberries) and vegetables (lettuce, spinach and onions) were sourced as study material from home gardens. Minimally processed leaf vegetables (baby spinach, iceberg lettuce, lamb’s lettuce and arugula) and stem, flower and root vegetables (leeks, broccoli and radishes) were also sourced as study samples from supermarkets. Food samples from home gardens were collected in sterile, tightly sealed bags, and products purchased from supermarkets were transported in the manufacturer’s original packaging. Samples were delivered to the laboratory at 4°C within 3 h. Immediately after arrival, they were prepared and microbiologically cultured. Additionally, north-eastern Polish farmland soil was sampled from the arable layer in autumn, before fertilisers were applied, and *Pantoea* strains were isolated from them. Samples were collected according to soil sampling guidelines, using a stick sampler, from a depth of up to 20 cm, with at least 10 penetrations to obtain an average probe. All produce was sampled in 2020–2022.

### Microbiological cultures

The research material was prepared by suspending 10 g of each sample in 90 mL of Ringer’s solution (Merck, Darmstadt, Germany) and homogenising for 4 min using a BagMixer 400SW blender (Interscience, Saint-Nom-la-Brétèche, France). Strain isolation was achieved by a dilution method on violet red bile glucose agar (VRBGA; Biomaxima, Lublin, Poland) for Gram-negative bacteria and nutrient agar (NA; Biomaxima) for mesophilic bacteria. Inoculated VRBGA medium was incubated at 37°C, and NA medium at 30°C, both for 24 h. Preliminary identification of *Pantoea* strains was based on macroscopic (colony shape and colour) and microscopic (cell shape and Gram-staining effect) observations.

### Biochemical identification

Strains were identified to genus level using biochemical tests: the ENTEROtest 24N product (Erba-Lachema, Brno, Czech Republic) and the GEN III MicroLog M System (Biolog, Hayward, CA, USA). The preparation of the inoculum and the performance of tests were carried out according to the manufacturer’s instructions. Interpretation of ENTEROtest 24N results was performed using a Multiskan EX automatic microplate reader (Thermo Fisher Scientific, Vantaa, Finland) and ErbaExpert software. MicroLog microplates were read using the GEN III MicroLog M system and the MicroLog M 5.2 program.

### Molecular analysis

The genomic DNA was isolated from the 24-h pure cultures of *Pantoea* strains on NA medium at 30°C using a QIAamp DNA Mini Kit (Qiagen, Hilden, Germany), according to the manufacturer’s protocol for Gram-negative bacteria. *Pantoea* species were detected and their diversity was characterised by amplification of 16S ribosomal RNA (rRNA) and *infB* (translation initiation factor 2), *atpD* (adenosine triphosphate synthase δ subunit), *gyrB* (DNA gyrase subunit B) and *rpoB* (RNA polymerase β subunit) gene fragments. Two efflux pump genes (*acrA* (anti– clustered regularly interspaced short palindromic repeat protein A) and *acrB*) were examined by single PCR reactions with the use of the American Tissue Culture Collection (ATCC) 25922 *Escherichia coli* strain as a positive control. Analysis of the type VI secretion system (T6SS) was performed for two effector proteins: Hcp (haemolysin-coregulated protein) and VgrG (glycine-valine repeat sequence G). A multiplex PCR using primer pairs for six families of Ambler class C (AmpC) β-lactamase–encoding genes determined these genes’ presence in *Pantoea* isolates: MOX (conferring resistance to moxalactam), CIT (originally characterised in *Citrobacter freundii*), DHA (characterised first in a sample in Dhahran Hospital), ACC (Ambler class C), EBC (originally characterised in *Enterobacter cloacae* complex) and FOX (conferring resistance to cefoxitin). Extended-spectrum βL–producing *Pantoea* isolates were examined for carriage of the SHV (sulfhydryl variant reagent) and TEM (patient Temoneira) genes. The primers, components and conditions of all mono- and multiplex PCR reactions performed in this study are listed in Tables 1 and 2. All PCR reactions were performed using a C1000 (BioRad, Hercules, CA, USA) or Mastercycler nexus (Eppendorf, Hamburg, Germany) thermal cycler. Amplification products were identified in 2% agarose gel (Prona Basica LE; Hispanagar/Prona Maritime Research Institute, Burgos, Spain) by horizontal electrophoresis under standard conditions (75 V and 400 mA for 55 min). After staining in 2 μg/mL ethidium bromide for 20 min, the amplification products were visualised using a gel documentation and image analysis system (InGenius LHR; Syngene, Cambridge, UK). The products were compared to a GeneRuler 100 bp Plus or 1 kb DNA Ladder (Thermo Fisher Scientific, Vilnius, Lithuania).

**Table 1.** Primers used in the study for molecular identification and assessment of virulence and drug resistance

	Primer	Target gene	Sequence (5′→3′)	Product size (bp)	Reference
For molecular identification	infB-01_F infB-02_R	*infB*	ATYATGGGHCAYGTHGAYCA ACKGAGTARTAACGCAGATCCA	1,124	(44)
	PANsp_atpD_F PANsp_atpD_F	*atpD*	GAGGGTAACGACTTCTACCAC CTGTACGGAGGTGATTGAAC	330	
	PANAG_infB_F PANAG_infB_R	*infB*	TGTCCGGCGTGCCGGCTG CCAACGCGAACGTCGTTGT	730	
	PANAN_gyrB_F PANAN_gyrB_R	*gyrB*	GATGACGARGCCATGCTGC GATCTTGCGGTATTCGCCAC	423	(28)
	PANST_rpoB_F PANST rpoB_R	*rpoB*	CACCGGTGAACTGATTATCG GTCCTGAGGCATCAATGTGT	539	
	16S_27F 16S907	16S rRNA	AGAGTTTGATCMTGGCTCAG CCCCGTCAATTCMTTTRAGTTT	920	
	p27_F pl525_R	16S rRNA	AGAGTTTGATCMTGGCTCAG AAGGAGGTGWTCCARCC	1,450	(30)
For virulence gene detection	acrA_F acrA_R	*acrA*	CTCTCAGGCAGCTTAGCCCTAA TGCAGAGGTTCAGTTTTGACTGTT	106	(31)
	acrB_F acrB_R	*acrB*	GGTCGATTCCGTTCTCCGTTA CTACCTGGAAGTAAACGTCATTGGT	104	
	hcp_F hcp_R	*hcp*	TGTAAACCAGCGCCATCAGT ACCGGTAATGCACAGCTGAA	1,301	(22)
	vgrG_F vgrG_R	*vgrG*	TGAATCCGCTTGCTTCCTGT ATATCGCCCATGCGTTCCAT	1,011	
For antimicrobial resistance gene detection	MOXM_F MOXM_R CITM_F CITM_R	*MOX-1, MOX-2, CMY-1, CMY-8-CMY-11, LAT-1-LAT-4, CMY-2-CMY-7, BIL-1*	GCTGCTCAAGGAGCACAGGAT CACATTGACATAGGTGTGGTGC TGGCCAGAACTGACAGGCAAA TTTCTCCTGAACGTGGCTGGC	520 462	
	DHAM_F DHAM_R	*DHA-1, DHA-2*	AACTTTCACAGGTGTGCTGGGT CCGTACGCATACTGGCTTTGC	405	(37)
	ACCM_F ACCM_R	*ACC*	AACAGCCTCAGCAGCCGGTTA TTCGCCGCAATCATCCCTAGC	346	
	EBCM_F EBCM_R	*MIR-1T, ACT-1*	TCGGTAAAGCCGATGTTGCGG CTTCCACTGCGGCTGCCAGTT	302	
	FOXM_F FOXM_R	*FOX-1-FOX-5b*	AACATGGGGTATCAGGGAGATG CAAAGCGCGTAACCGGATTGG	190	
	SHV_F SHV_R	*SHV*	CACTCAAGGATGTATTGTG TTAGCGTTGCCAGTGCTCG	885	(26)
	TEM-1_F TEM-1 R	*TEM*	AAGCCATACCAAACGACGAG ATTGTTGCCGGGAAGCTAGA	108	(27)

1 F – forward; R – reverse; *infB* – translation initiation factor 2; *atpD* – adenosine triphosphate synthase δ subunit; *gyrB* – DNA gyrase subunit B; *rpoB* – RNA polymerase β subunit; rRNA – ribosomal RNA; *acrA/B* – anti–clustered regularly interspaced short palindromic repeat protein A/protein B; *hcp* – haemolysin-coregulated protein; *vgrG* – glycine-valine repeat sequence G; *MOX* – conferring resistance to moxalactam; *CMY* – conferring resistance to cephamycin; *LAT* – conferring resistance to latamoxef/moxalactam; *BIL* – patient Bilal; *DHA* –Dhahran Hospital; *ACC* – Ambler class C; *MIR* – Miriam Hospital; *ACT* – Ambler class C type; *FOX* – conferring resistance to cefoxitin; *SHV* – sulfhydryl variant reagent; *TEM* – patient Temoneira

**Table 2.** Reaction mixtures and conditions of PCR reactions performed in the study

Gene(s)	Total volume	PCR reaction mixture	Initial denaturation (temp./duration)	Denaturation, annealing, extension (temp./duration)	Number of cycles	Final extension (temp./duration)
		Reagents				
*infB* ^1^	25 μL	0.3 μL Taq DNA Polymerase (5U/μL)^a^ 2.5 μL 10× PCR buffer with 15 mM MgCl_2_^b^ 2.5 μL 2 mM dNTPs^c^ 1 μL 10 pmol of each primer^d^ 2.5 μL DNA template 15.2 μL nuclease-free water^e^	95°C/5 min	94°C/45 s 55°C/1 min 72°C/1.5 min	30	72°C/7 min
*atpD, infB, gyrB, rpoB*, 16S rRNA^2^	25 μL	0.2 μL Taq DNA Polymerase (5U/μL) 2.5 μL 10× PCR buffer with 15 mM MgCl_2_ 2.5 μL 2 mM dNTPs 0.5 μL DNA template 12.8 μL nuclease-free water	94°C/3 min	94°C/3 min 94°C/30 s 58°C/30 s 72°C/2 min	30	72°C/10 min
16S rRNA^1^	50 μL	0.3 μL Taq DNA Polymerase (5U/μL) 5 μL 10× PCR buffer with 15 mM MgCl_2_ 5 μL 2 mM dNTPs 2 μL 10 pmol of each primer 5 μL DNA template 30.7 μL nuclease-free water	94°C/2 min	94°C/1 min 55°C/1 min 72°C/3 min	25	72°C/10 min
*acrA* or *acrB*^1^	20 μL	0.16 μL Taq DNA Polymerase (5U/μL) 2.5 μL 10× PCR buffer with 15 mM MgCl_2_ 2.5 μL 2 mM dNTPs 0.5 μL 10 pmol of each primer 2 μL DNA template 12.34 μL nuclease-free water	95°C/5 min	95°C/30 s 56°C/30 s(*acrA*) and 55°C/30 s (*acrB*) 72°C/2 min	30	72°C/10 min
*hcp* or *vgrG*^1^ ESβL genes^1^	25 μL	0.2 μL Taq DNA Polymerase (5U/μL) 2.5 μL 10× PCR buffer with 15 mM MgCl_2_ 2.5 μL 2 mM dNTPs 1 μL 10 pmol of each primer 2 μL DNA template 15.8 μL nuclease-free water	95°C/5 min 96°C/1 min	95°C/30 s 55°C/30 s 72°C/2 min 94°C/1 min 50°C/1 min 72°C/2 min	25 35	72°C/10 min 72°C/10 min
AmpC genes^2^	25 μL	0.2 μL Taq DNA Polymerase (5U/μL) 2.5 μL 10× PCR buffer with 15 mM MgCl_2_ 2.5 μL 2 mM dNTPs 1 μL 10 pmol of each primer 2 μL DNA template 5.8 μL nuclease-free water	94°C/3 min	94°C/30 s 64°C/30 s 72°C/1 min	25	72°C/10 min

1 Reagents: Taq DNA Polymerase (Qiagen, Hilden, Germany), 10×PCR buffer containing 15 mM MgCl_2_ (Qiagen), dNTP Mix (deoxyribonucleoside triphosphate – Thermo Fisher Scientific, Vilnius, Lithuania), primers (Institute of Biochemistry and Biophysics, Warsaw, Poland), nuclease-free water (Qiagen). *infB* – translation initiation factor 2; *atpD* – adenosine triphosphate synthase δ subunit; *gyrB* – DNA gyrase subunit B; *rpoB* – RNA polymerase β subunit; rRNA – ribosomal RNA; *acrA/B* – anti–clustered regularly interspaced short palindromic repeat protein A/protein B; *hcp* – haemolysin-coregulated protein; *vgrG* – glycine-valine repeat sequence G; ESβL – extended-spectrum β-lactamase; AmpC – Ambler class C;

1 – monoplex PCR;

2 – multiplex PCR

Sequencing was performed with an ABI PRISM 310 Genetic Analyzer (Applied Biosystems, Waltham, MA, USA) using ABI PRISM Big Dye Terminator v. 3.1. Cycle Sequencing Kits and a Big Dye XTerminator Purification Kit (Applied Biosystems). The results of the nucleotide sequences were compared with data stored in the GenBank database using the Basic Local Alignment Search Tool software at the National Centre for Biotechnology Information (Bethesda, MD, USA). The sequences obtained in the current study were deposited in the GenBank database.

## Assessment of enzymatic activities

Enzymatic activities were detected using the API ZYM semi-quantitative method (bioMérieux, Marcy-l’Étoile, France) allowing the analysis of 19 specific enzymes. A cell suspension with a density of 5–6 McFarland was prepared from the culture of *Pantoea* strains obtained after 24-h incubation on NA medium. The test procedure was performed according to the manufacturer’s instructions. Enzyme activity was determined in nanomoles of the hydrolysed substrate according to Bang and Chung (4).

## Assessment of antibiotic resistance

A standardised bacterial inoculum (0.5 McFarland) was spread on the surface of Mueller–Hinton Agar (MHA) plates (Biomaxima). Antibiotic susceptibility testing was performed using the Kirby–Bauer disc-diffusion method. Antibiotic discs (Biomaxima) were placed on MHA plates and incubated at 35°C for 18 h. The diameter of the inhibition zones was measured in millimetres. The minimum inhibitory concentration (MIC) was determined using MIC G-I and MIC G-II kits (Erba-Lachema). Bacterial suspensions were prepared from 24-h cultures on blood agar (Biomaxima) at 37°C, and the optical density was adjusted to 0.5 McFarland. Then, 60 μL of the prepared bacterial suspension was mixed with 13 mL of Mueller Hinton II broth, and 100 μL of this inoculated broth was added to the test wells containing the appropriate concentrations of the tested antibiotics (Supplementary Table S1). An *Escherichia coli* strain (ATCC 25922) was used as a quality control. Results were interpreted using the Multiskan EX automated reader and ErbaExpert software. The resistance profiles of isolates obtained by both methods were determined based on the recommendations of the Clinical and Laboratory Standards Institute (13) for kanamycin and streptomycin and the European Committee on Antimicrobial Susceptibility Testing (17) for other antimicrobial agents.

## Results

### Microbiological and biochemical identification

Based on macro- and microscopic observation of bacterial colonies, 30 strains were qualified for further analysis. Yellow bacterial colonies were isolated from a 24-h culture. Preparations made with Gram staining showed the presence of Gram-negative bacteria with rod-shaped morphology. Preliminary identification using the ENTEROtest 24N test and the GEN III MicroLog M System indicated that all strains belonged to the *Pantoea* genus. Combined, the tests confirmed the species identification of 7 strains: 5 of *P. agglomerans* and 2 of *P. ananatis*. In two cases, the ENTEROtest 24N test yielded results for *P. agglomerans*, while the Gen III Micro-log M System method confirmed only the isolates’ genus affiliation (Table 3). All strains produced acids from trehalose, mannitol and sucrose. *Pantoea ananatis* strains were the only ones that showed ß-xylosidase activity and the ability to produce acid from sorbitol, cellobiose and lactose. None of the strains showed urease or β-glucuronidase activity or produced hydrogen sulphide, decarboxylated ornithine or arginine. The strains also did not produce acid from dulcitol or adonitol (Supplementary Fig. S1). All strains showed bacterial growth in the presence of 1% and 5% NaCl, but none in the presence of 8% NaCl. All isolated strains were able to grow at pH 5–6 and in the presence of 1% sodium lactate.

**Table 3.** Results of biochemical and molecular identification of *Pantoea* strains isolated from food and soil

Strain No.	Sample type	Biochemical identification	Molecular identification						Sequence match
			*infB* (*Panyoea* spp.)	*infB* (*P. agglomerans*)	*gyrB* (*P. ananatis*)	*rpoB* (*P. stewartii*)	*atpD* (*Pantoea* spp.)	16S (Eubacteria)	
1S	strawberry	*P. agglomerans*	+	+	–	–	+	+	*P. agglomerans*
2S	raspberry	*Pantoea* spp.	+	+	–	–	+	+	*P. agglomerans*
3S	lettuce	*Pantoea* spp.	+	+	–	–	+	+	*P. agglomerans*
4S	spinach	*P. agglomerans*	+	+	–	–	+	+	*P. agglomerans*
5S	baby spinach	*Pantoea* spp.	+	+	–	–	+	+	*P. agglomerans*
6S	arugula	*Pantoea* spp.	+	+	–	–	+	+	*P. agglomerans*
7S	onion	*P. agglomerans*	+	+	–	–	+	+	*P. agglomerans*
8S	baby spinach	*Pantoea* spp.	–	–	–	–	+	+	*P. eucalypti*
9S	iceberg lettuce	*Pantoea* spp.	+	–	–	–	+	+	*P. conspicua*
10S	lam’s lettuce	*Pantoea* spp.	+	+	–	–	+	+	*P. agglomerans*
11S	soil	*Pantoea* spp.	+	+	–	–	+	+	*P. agglomerans*
12S	soil	*Pantoea* spp.	+	+	–	–	+	+	*P. agglomerans*
13S	soil	*Pantoea* spp.	–	+	–	–	+	+	*P. agglomerans*
14S	soil	*Pantoea* spp.	+	+	–	–	+	+	*P. agglomerans*
15S	soil	*P. agglomerans*	+	+	–	–	+	+	*P. agglomerans*
16S	soil	*Pantoea* spp.	+	+	–	–	+	+	*P. agglomerans*
17S	soil	*Pantoea* spp.	+	+	–	–	+	+	*P. agglomerans*
18S	soil	*Pantoea* spp.	+	+	–	–	+	+	*P. agglomerans*
19S	soil	*Pantoea* spp.	+	+	–	–	+	+	*P. agglomerans*
20S	soil	*Pantoea* spp.	+	+	–	–	+	+	*P. agglomerans*
21S	soil	*P. agglomerans*	+	+	–	–	+	+	*P. agglomerans*
22S	soil	*Pantoea* spp.	+	+	–	–	+	+	*P. agglomerans*
23S	soil	*P. agglomerans*	+	+	–	–	+	+	*P. agglomerans*
24S	soil	*Pantoea* spp.	+	+	–	–	+	+	*P. agglomerans*
25S	leek	*P. ananatis*	+	–	+	–	–	+	*P. ananatis*
26S	radish	*P. agglomerans*	+	+	–	–	+	+	*P. agglomerans*
27S	broccoli	*Pantoea* spp.	–	–	–	–	+	+	*P. vagans*
28S	soil	*Pantoea* spp.	+	+	–	–	+	+	*P. agglomerans*
29S	soil	*Pantoea* spp.	–	–	–	–	+	+	*P. vagans*
30S	leek	*P. ananatis*	+	–	+	–	–	+	*Pantoea sp*.

1 *infB* – translation initiation factor 2; *gyrB* – DNA gyrase subunit B; *rpoB* – RNA polymerase β subunit; *atpD* – adenosine triphosphate synthase *δ* subunit

### Molecular identification

Sequencing based on 16S rRNA gene fragments confirmed the identification of 29 strains: 24 to the species *P. agglomerans*; 1 each to *P. ananatis, P. eucalypti* and *P. conspicua*; and 2 to *P. vagans*. In one case, the *P. ananatis* strain identified in the biochemical tests was not confirmed (Table 3).

**Fig. 1.**
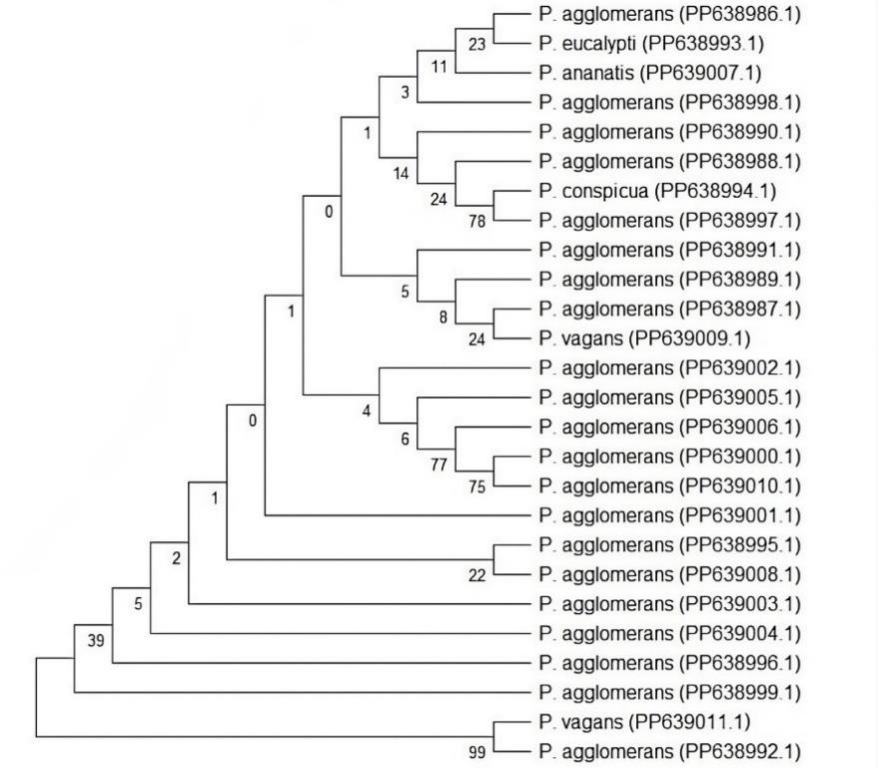
Phylogenetic tree based on the partial 16S rRNA gene sequences of *Pantoea* strains isolated from food and soil

The obtained sequences of 26 strains of appropriate length were deposited in the GenBank database, and then an evolutionary analysis of the taxa was performed on a phylogenetic tree (Fig. 1).

### Virulence gene occurrence

The *hcp* gene was detected in 13 *P. agglomerans* strains, 2 *P. ananatis* strains and 2 *P. vagans* strains. In one strain of *P. agglomerans*, the presence of both effector proteins, *hcp* and *vgrG*, was demonstrated. The *acrA* and *acrB* pump genes were not detected in any of the strains tested (Table 4).

**Table 4.** Occurrence of virulence and drug resistance genes in *Pantoea* strains

Species (strain No.)	Sample type	Virulence factor				Drug resistance gene							
		*acrA*	*acrB*	*vgrG*	*hcp*	*TEM*	*FOX*	*SHV*	*MOX*	*CIT*	*DHA*	*ACC*	*EBC*
*P. agglomerans* (26S)	radish	–	–	+	+	+	+	–	–	–	–	–	–
*P. agglomerans* (1S)	raspberry	–	–	–	+	+	+	–	–	–	–	–	–
*P. agglomerans* (6S)	arugula	–	–	–	+	+	+	–	–	–	–	–	–
*P. agglomerans* (10S)	lamb’s lettuce	–	–	–	+	+	+	–	–	–	–	–	–
*P. agglomerans* (13S)	soil	–	–	–	+	+	+	–	–	–	–	–	–
*P. agglomerans* (17S)	soil	–	–	–	+	+	+	–	–	–	–	–	–
*P. agglomerans* (20S)	soil	–	–	–	+	+	+	–	–	–	–	–	–
*P. agglomerans* (21S)	soil	–	–	–	+	+	+	–	–	–	–	–	–
*P. agglomerans* (23S)	soil	–	–	–	+	+	+	–	–	–	–	–	–
*P. agglomerans* (28S)	soil	–	–	–	+	+	+	–	–	–	–	–	–
*P. vagans* (29S)	soil	–	–	–	+	+	+	–	–	–	–	–	–
*P. agglomerans* (22S)	soil	–	–	–	+	+	–	+	–	–	–	–	–
*P. agglomerans* (3S)	lettuce	–	–	–	+	–	–	–	–	–	–	–	–
*P. agglomerans* (12S)	soil	–	–	–	+	+	–	–	–	–	–	–	–
*P. ananatis* (25S)	leek	–	–	–	+	+	–	–	–	–	–	–	–
*P. vagans* (27S)	broccoli	–	–	–	+	+	–	–	–	–	–	–	–
*P. ananatis* (30S)	leek	–	–	–	+	+	–	–	–	–	–	–	–
*P. agglomerans* (4S)	spinach	–	–	–	–	+	+	–	–	–	–	–	–
*P. agglomerans* (5S)	baby spinach	–	–	–	–	+	+	–	–	–	–	–	–
*P. eucalypti* (8S)	baby spinach	–	–	–	–	+	+	–	–	–	–	–	–
*P. agglomerans* (11S)	soil	–	–	–	–	+	+	–	–	–	–	–	–
*P. agglomerans* (15S)	soil	–	–	–	–	+	+	–	–	–	–	–	–
*P. agglomerans* (18S)	soil	–	–	–	–	+	+	–	–	–	–	–	–
*P. agglomerans* (7S)	onion	–	–	–	–	+	–	+	–	–	–	–	–
*P. conspicua* (9S)	iceberg lettuce	–	–	–	–	+	–	+	–	–	–	–	–
*P. agglomerans* (2S)	raspberry	–	–	–	–	–	+	–	–	–	–	–	–
*P. agglomerans* (16S)	soil	–	–	–	–	–	+	–	–	–	–	–	–
*P. agglomerans* (19S)	soil	–	–	–	–	–	+	–	–	–	–	–	–
*P. agglomerans* (24S)	soil	–	–	–	–	–	+	–	–	–	–	–	–
*P. agglomerans* (14S)	soil	–	–	–	–	+	–	–	–	–	–	–	–

1 *acrA/B* – anti–clustered regularly interspaced short palindromic repeat protein A/protein B; *vgrG* – glycine-valine repeat sequence G; *hcp* – haemolysin-coregulated protein; *TEM* – patient Temoneira; *FOX* – conferring resistance to cefoxitin; *SHV* – sulfhydryl variant reagent; *MOX* – conferring resistance to moxalactam; *CIT* – originally characterised in *Citrobacter freundii*; *DHA* – Dhahran Hospital; *ACC* – Ambler class C; *EBC* – originally characterised in *Enterobacter cloacae* complex

### Drug resistance gene prevalence

The presence of AmpC β-lactamase–resistance genes was confirmed in 22 strains. The presence of *FOX* family genes was identified in 22 isolates belonging to the *P. agglomerans, P. eucalypti* and *P. vagans* species. No genes associated with the *MOX, CIT, DHA, ACC* or *EBC* families were identified. Detection of the *TEM* and *SHV* genes was achieved in 25 and 3 *Pantoea* strains, respectively (Table 4). The prevalence of ESβL-producing isolates was 83.3%, but only three isolates had both ESβL genes – two *P. agglomerans* and one *P. conspicua* strain.

### Enzymatic activity

**Fig. 2.**
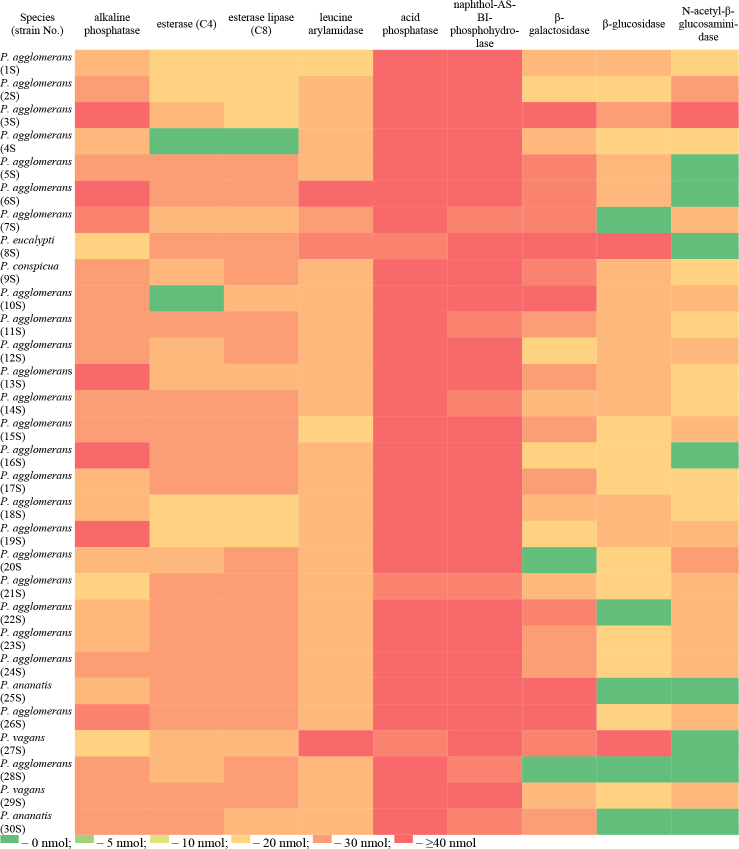
The activity of hydrolytic enzymes of *Pantoea* strains isolated from food and soil

Of all the enzymes tested, the highest activity was demonstrated by phosphatases, such as acid phosphatase and naphthol-AS-BI phosphohydrolase. In addition to phosphatase activity, high activity of hydrolytic esterases (esterase-lipase and esterase), amino-peptidases (leucine arylamidase) and glycosylhydrolases (β-galactosidase, β-glucosidase and N-acetyl-β-glucosaminidase) was discovered. The lack of activity of lipase (C14), valine arylamidase, cystine arylamidase, trypsin, α-chymotrypsin, α-galactosidase, β-glucuronidase, α-glucosidase, α-mannosidase and α-fucosidase was noted in all the *Pantoea* strains (Fig. 2).

### Drug resistance

Most of the tested *Pantoea* strains isolated from food and soil showed susceptibility to the antimicrobial agents used in the study. Intermediate or complete resistance to nine (45%) antimicrobial agents used in the disc-diffusion test (Supplementary Table S2) was shown by at least one of the strains isolated from food. However, only two strains isolated from food (10%) showed intermediate or complete resistance. Similar results were obtained for resistance to antimicrobial agents used in the concentration gradient method (Supplementary Table S3). Nine (38%) antimicrobial agents were resisted by at least one of the strains isolated from food, while four failed to prevent the growth of strains isolated from soil.

All strains (100%) showed high resistance to cephazolin and cephuroxime. More than half (53.3%) also showed resistance to ampicillin. Antimicrobial susceptibility testing revealed the presence of multidrugresistant (MDR) strains isolated from both food (14.3%) and soil (6.3%), including *P. agglomerans* isolated from arugula (resistant to a monobactam, amphenicol, fluoroquinolone, tetracycline, penicillin and cephalosporin) and soil (resistant to penicillin, cephalosporin and tetracycline), *P. ananatis* isolated from leeks (resistant to carbapenem, penicillin, cephalosporin, polymyxin).

## Discussion

To date, more than 20 species belonging to the *Pantoea* genus have been identified, including clinically important ones such as *P. agglomerans, P. ananatis* and *P. conspicua* (16, 29, 32). The results obtained in this study confirmed that the *Pantoea* strains isolated from food and soil belonged to five species: *P. agglomerans, P. eucalypti, P. ananatis, P. conspicua* and *P. vagans*. Species identification was possible using molecular studies, while biochemical results mainly allowed genus identification (46). The combined use of both methods appears to be most effective in identifying bacteria to species level, whereby initial identification using biochemical tests can be helpful in selecting appropriate genetic methods. Biochemical analyses revealed differences between the identified species, such as that between *P. ananatis* and the other strains tested in terms of the utilisation of sorbitol, lactose and cellobiose as carbohydrate sources and the ability to produce β-xylosidase. Studies by Chukwuma *et al*. (12) also confirmed differentiating characteristics of *P. ananatis* and *P. agglomerans*, including the ability to utilise melibiose and indole. The identified *Pantoea* strains showed the activity of nine enzymes, with the highest activity noted for phosphatases. Hydrolytic enzymes produced by bacteria facilitate colonisation and invasion of their associated host by providing nutrients necessary for growth, modifying the local environment to provide suitable conditions, and assisting in metabolism (15).

Higher species identification efficiency was obtained using the multiplex PCR method developed by Kini *et al*. (28) than with the monoplex PCR proposed by Shin *et al*. (44). Amplification of an *atpD* gene fragment in a single PCR reaction targeted a more specific site in the bacterial DNA, typical of the *Pantoea* genus, than amplification of the *infB* gene could. However, amplification of an *infB* gene fragment in the multiplex PCR proved effective in identifying the *P. agglomerans* species (28). The host gene sequences used in this study allowed the correct identification of *P. agglomerans* and *P. ananatis*, confirmed by sequencing. For the remaining species (*P. eucalypti, P. conspicua* and *P. vagans*), identification was possible only based on sequencing results.

Literature data show that the most frequently isolated *Pantoea* species from fresh or minimally processed food and from soil are *P. agglomerans* and *P. ananatis* (34, 46), and less often *P. conspicua, P. vagans* and *P. eucalypti* are recovered (6). Among these *Pantoea* spp. are bacteria which can cause opportunistic infections in humans, as evidenced by their presence in clinical material (6, 34). Therefore, contaminated food may pose a health risk, especially to immunocompromised individuals. However, scientific reports in this area are still scarce. The source of food plant infection with *Pantoea* may be soil, where the presence of live bacteria, primarily *P. agglomerans*, has been confirmed in the current research. Studies by Cernava *et al*. (11) confirmed that these bacteria can be transferred from soil to various plant tissues during plant growth.

Determining virulence factors and drug resistance in environmental *Pantoea* strains, which cause infections mainly in tissue through penetrating trauma or in the respiratory system through inhalation (7, 36, 46), may be crucial for the proper treatment of these infections in humans. Molecular analysis of virulence factors of *Pantoea* strains led to the identification of genes related to T6SS. One of them is *hcp*, the product of which, haemolysin-coregulated protein, forms a hexameric ring. Genes encoding this protein were identified in *Salmonella typhimurium*, and their role in bacterial competition, bacterial motility and long-term intracellular survival in eukaryotic cells, and their probable involvement in the early stage of bacterial infection were reported (49). Wang *et al*. (48) also confirmed the presence of these genes in *Aeromonas hydrophila*, indicating their involvement in various processes of adaptation to the environment and in bacterial virulence, including adhesion or biofilm formation. In the present study, *hcp* was identified in *P. agglomerans* and *P. ananatis* strains. The presence of these genes was also confirmed by other scientists in other food-borne isolates of *P. agglomerans, P. ananatis* and *P. vagans* (22, 43).

The *vgrG* gene was identified in one food-derived *P. agglomerans* strain. Proteins of VgrG assemble into a trimeric complex structurally resembling the cellpuncturing spike complex used by T4 bacteriophages. These proteins, similarly to Hcp, can perform various functions, reflecting the evolutionary diversity of VgrG C-terminal domains. It was confirmed that the evolved VgrG proteins in *Vibrio cholerae* carry the actin crosslinking domain on the C-terminal extension, which determines cytotoxicity towards host cells (38). Evolved vgrG proteins may be involved in cell adhesion, degradation of the peptidoglycan layer, ADP-ribosylation of host proteins and the transport of toxins (5, 38). Research conducted by De Maayer *et al*. (14) indicated the occurrence of diverse C-terminal domains in *Pantoea* strains, including the presence of a peptidoglycan-binding domain or a lysozyme domain.

The present study examined the occurrence of genes encoding components of the AcrAB complex that play a specific role in environmental adaptation and microbial virulence (18). Multidrug efflux pumps, such as the AcrAB-tolC efflux pump, are clinically important mechanisms driving resistance to antibiotics used to treat bacterial infections in humans (24). The presence of the AcrAB-tolC efflux pump has been detected in several species of the *Enterobacteriaceae* family, such as *Escherichia coli* or *Klebsiella pneumonia* (18, 40). Given that *Pantoea* was classified within this family until recently, similar efflux mechanisms might be expected in *Pantoea* species. However, the present study did not demonstrate the presence of *acrA* or *acrB* in *Pantoea* spp. isolated from food and soil. Similar results were also obtained by Abdulamir *et al*. (1), who failed to detect these genes in either clinical or environmental *Pantoea* isolates. However, different results were obtained in the study by Fernández Fuentes *et al*. (19), in which *P. agglomerans* and *P. ananatis* isolated from food both harboured the *acrB* gene.

A growing number of reports indicate the interplay of virulence and increasing antibiotic resistance among microorganisms, including bacteria from the Enterobacterales order (40). Both the World Health Organization and the Centers for Disease Control and Prevention (CDC) have raised concerns over pathogenic antimicrobial-resistant *Enterobacterales* (10, 47). The drug resistance findings for *Pantoea* strains obtained in this study confirm their ability to produce ESβL, which confer resistance to cephalosporins and β-lactam antibiotics. Although these bacteria are not directly mentioned in the 2019 and 2022 CDC reports, both reports indicate an alarming increase in the number of ESβL-producing *Enterobacterales* (9, 10). Of particular concern is the high level of resistance to cephazolin and cephuroxime found among all *Pantoea* strains isolated in this study. Raphael *et al*. (39), examining the level of resistance to these cephalosporins among *P. agglomerans* and *P. ananatis* isolated from spinach, showed a much lower level of resistance, amounting to 53% and 8% to cephazolin and 27% and 0% to cephuroxime, respectively. In turn, Mwangi *et al*. (34) showed 100% resistance to cephazolin and cephuroxime among *Pantoea* strains isolated from the urine of patients admitted to an intensive care unit.

The presence of genes encoding ESβL and/or AmpC, which hydrolyse the β-lactam antibiotics used for many infections caused by Gram-negative bacteria, was confirmed in *Pantoea* spp. isolated from soil and food. Most of the tested strains showed the presence of the *TEM* gene (83%), while only three strains (10%) showed the presence of the *SHV* gene. However, some studies did not show the presence of either gene in *Pantoea* spp. isolated from fresh and ready-to-eat vegetables (8, 25) or did not show the presence of both genes in the same strain among such isolates (23). In turn, the results obtained in the current study regarding the frequency of these genes are similar to the results of the study conducted by Alrufaie *et al*. (3), in which most tested *Pantoea* isolates from urine carried *TEM* (78.5%), and a sizeable proportion had *SHV* (28.6%). The present study also demonstrated the presence of the *FOX* gene in the tested strains (73%). To our knowledge, this is the first report indicating the presence of *FOX* in *Pantoea* bacteria isolated from food and soil. Research conducted by Iseppi *et al*. (23) did not find it in *P. agglomerans* strains isolated from fresh vegetables and mixed salad. The presence of the *FOX* gene in *Pantoea* strains is clinically important because it confers resistance to the cephalosporins and cephamycins used to treat a wide range of infections caused by Gram-negative bacteria (3).

The tests conducted showed the presence of three MDR *Pantoea* strains. To our knowledge, the current study is one of not many indicating the presence of multidrug-resistant *Pantoea* strains in food and soil. The presence of MDR *Pantoea* spp. was confirmed in kimchi (22) and powdered infant formula milk (33). Currently, MDR *Pantoea* strains resistant to new antimicrobials are increasingly frequently encountered in clinical practice. The gravity of the risk of therapeutic ineffectivity is indicated by the account of Büyükcam *et al*. (7) of three fatal cases in patients with infection caused by multidrugresistant strains of *P. agglomerans*. According to previous literature reports on the development of infections caused by various *Pantoea* species and the patients’ significant improvement after the use of appropriate antimicrobial agents, early recognition of the disease is important, as well as selection of the appropriate therapy that can prevent the invasion of potential pathogens (9, 10).

## Conclusion

Fresh vegetables, fruits and soil can be sources of infections with bacteria from the *Pantoea* genus. Among the isolated strains, *P. agglomerans* was found to be the dominant species. In addition to the identified virulence factors, such as T6SS elements that enable infection initiation, three antimicrobial resistance genes were detected in the tested strains. The resistance results indicate higher antimicrobial resistance among *Pantoea* strains isolated from food compared to resistance in strains isolated from soil. Knowledge of *Pantoea* spp. at the molecular and biochemical level gained from the determination of drug resistance and identification of virulence factors may be crucial in the clinical treatment of infections in humans.

## Supplementary Material

Supplementary Material Details
