# Supplementary material for: Genetic diversity, virulence factors and drug resistance of Pantoea strains isolated from samples of fresh fruits, vegetables and soil
Source: J Vet Res. 2025 Sep 13;69(3):477–88. doi: 10.2478/jvetres-2025-0047 (PMC12503223; doi:10.2478/jvetres-2025-0047)
Supplement: Supplementary file 1 — Supplementary Material Details [file jvetres-2025-0047_sm.pdf]

Supplementary Table S1. List of antimicrobial drugs used in the study

Drug name	Year of approval	Class	Method (concentration in antibiotic disc or range of MIC values)
Amikacin	1976	Aminoglycosides	MIC determination (0.5–64 mg/L)
Amoxicillin/clavulanic acid	1981	Penicillins	disc-diffusion method (20/10 µg)
Ampicillin	1961	Penicillins	disc-diffusion method (10 µg)<br>MIC determination (1–128 mg/L)
Ampicillin/sulbactam	1987	Penicillins	disc-diffusion method (10/10 µg)<br>MIC determination (1/0.5–128/64 mg/L)
Azithromycin	1988	Macrolides	disc-diffusion method (15 µg)
Aztreonam	1986	Monobactams	disc-diffusion method (30 µg)<br>MIC determination (0.12–16 mg/L)
Cephazolin	1971	Cephalosporins	MIC determination (0.12–16 mg/L)
Cephepime	1994	Cephalosporins	MIC determination (0.12–16 mg/L)
Cephoperazone	1981	Cephalosporins	MIC determination (0.5–64 mg/L)
Cephoperazone/sulbactam	1986	Cephalosporins	MIC determination (0.5/0.25–64/32 mg/L)
Cephotaxime	1980	Cephalosporins	MIC determination (0.06–128 mg/L)
Cephtazidime	1984	Cephalosporins	disc-diffusion method (30 µg)<br>MIC determination (0.12–16 mg/L)
Cephuroxime	1977	Cephalosporins	disc-diffusion method (30 µg)<br>MIC determination (0.5–64 mg/L)
Chloramphenicol	1948	Amphenicols	disc-diffusion method (30 µg)<br>MIC determination (0.25–32 mg/L)
Ciprofloxacin	1987	Fluoroquinolones	MIC determination (0.06–8 mg/L)
Colistin	1959	Polymyxins	MIC determination (0.12–16 mg/L)
Doxycycline	1967	Tetracyclines	disc-diffusion method (30 µg)
Ertapenem	2001	Carbapenems	MIC determination (0.015–2 mg/L)
Gentamicin	1971	Aminoglycosides	MIC determination (0.25–32 mg/L)
Imipenem	1985	Carbapenems	disc-diffusion method (10 µg)
Kanamycin	1999*	Aminoglycosides	disc-diffusion method (30 µg)
Meropenem	1995	Carbapenems	disc-diffusion method (10 µg)<br>MIC determination (0.12–16 mg/L)
Nalidixic acid	1692	Fluoroquinolones	disc-diffusion method (30 µg)
Netilmicin	1981	Aminoglycosides	MIC determination (0.12–16 mg/L)
Ofloxacin	1990	Fluoroquinolones	disc-diffusion method (5 µg)
Piperacillin	1981	Penicillins	disc-diffusion method (100 µg)<br>MIC determination (1–128 mg/L)
Piperacillin/tazobactam	1993	Penicillins	disc-diffusion method (100/10 µg)<br>MIC determination (1/4–128/4 mg/L)
Streptomycin	1946	Aminoglycosides	disc-diffusion method (10 µg)
Sulphamethoxazole/trimethoprim	1986	Sulphonamides	disc-diffusion method (23.75/1.25 µg)<br>MIC determination (0.03/0.6–4/76 mg/L)
Tetracycline	1954	Tetracyclines	disc-diffusion method (30 µg)<br>MIC determination (0.25–32 mg/L)
Ticarcillin/clavulanate	1985	Penicillins	disc-diffusion method (75/10 µg)
Tigecycline	2005	Tetracyclines	MIC determination (0.06–8 mg/L)
Tobramycin	1974	Aminoglycosides	MIC determination (0.12–8 mg/L)

* – withdrawn from the market in 2019. MIC – minimum inhibitory concentration

Supplementary Table S2. Susceptibility profiles to antimicrobial agents used in the disc-diffusion method

Antimicrobial agent	Zone of inhibition (mm)		Susceptibility (n)					
	Food isolates	Soil isolates	Food isolates			Soil isolates		
			R	I	S	R	I	S
Aztreonam	34.1 (13–45)	37.8 (30–45)	1	0	13	0	0	16
Chloramphenicol	31.1 (17–38)	34.3 (32–38)	1	0	13	0	0	16
Ofloxacin	35.0 (22–40)	36.7 (30–40)	0	0	14	0	0	16
Nalidixic acid	31.6 (13–35)	31.9 (27–35)	1	0	13	0	0	16
Azithromycin	28.9 (21–35)	28.9 (27–35)	0	0	14	0	0	16
Doxycycline	24.7 (13–30)	25.2 (22–30)	0	2	12	0	0	16
Kanamycin	28.6 (22–35)	29.1 (25–35)	0	0	14	0	0	16
Streptomycin	23.0 (18–26)	23.4 (20–26)	0	0	14	0	0	16
Amoxicillin/clavulanic acid	22.8 (12–28)	23.1 (11–28)	1	1	12	1	1	14
Imipenem	30.9 (19–32)	29.15 (25–32)	1	0	13	0	0	16
Piperacillin/tazobactam	29.8 (23–37)	31.6 (26–37)	0	0	14	0	0	16
Trimethoprim/ sulfamethoxazole	38.1 (21–45)	39.5 (32–45)	0	0	14	0	0	16
Ampicillin/sulbactam	24.8 (12–30)	25.7 (22–30)	0	1	13	0	0	16
Ceftazidime	31.2 (27–38)	31.3 (26–38)	0	0	14	0	0	16
Meropenem	36.4 (32–42)	38.5 (35–42)	0	0	14	0	0	16
Tigecycline	29.6 (24–33)	29.7 (27–33)	0	0	14	0	0	16
Ticarcillin/clavulanic acid	31.9 (25–37)	31.6 (20–37)	0	0	14	0	0	16
Ampicillin	20.0 (11–27)	21.7 (14–27)	3	0	11	0	3	16
Cefuroxime	26.8 (9–36)	29.1 (23–36)	1	0	13	0	0	16
Piperacillin	30.3 (26–35)	31.5 (26–35)	0	0	14	0	0	16

S – susceptible; R – resistant; I – intermediate

Supplementary Table S3. Antimicrobial susceptibility profiles obtained based on MIC values

Antimicrobial agent	MIC/MIC range (mg/L)		Susceptibility (n)					
	Food isolates	Soil isolates	Food isolates			Soil isolates		
			R	I	S	R	I	S
Amikacin	≤0.500	≤0.500	0	0	14	0	0	16
Ampicillin	4–32	4–32	9	0	5	7	0	9
Ampicillin/sulbactam	≤1–32	2–8	3	0	11	0	0	16
Aztreonam	≤0.125–8	≤0.125–0.25	1	0	13	0	0	16
Cephazolin	4–>16	4->16	11	3	0	13	3	0
Cephuroxime	2–>64	2–8	1	13	0	0	16	0
Chloramphenicol	0.5–16	0.5–2	1	0	13	0	0	16
Ciprofloxacin	≤0.060–0.12	≤0.060	0	0	14	0	0	16
Gentamicin	≤0.250–0.5	≤0.250–0.5	0	0	14	0	0	16
Colistin	0.25–>16	0.25–1	2	0	12	0	0	16
Trimethoprim/sulphamethoxazole	≤0.030–1	≤0.030–0.06	0	0	14	0	0	16
Tetracycline	≤0.500–1	≤0.500–1	0	0	14	0	0	16
Cephepime	≤0.120–2	≤0.120–0.25	0	1	13	0	0	16
Cephoperazone	≤0.500–4	≤0.500–2	0	0	14	0	0	16
Cephoperazone/ sulbactam	≤0.500–4	0.500–1	0	0	14	0	0	16
Cephotaxime	≤0.060–8	≤0.060	1	1	12	0	0	16
Cephtazidime	≤0.120–1	≤0.120–1	0	0	14	0	0	16
Ertapenem	≤0.015–0.03	≤0.015–0.03	0	0	14	0	0	16
Meropenem	≤0.120–0.5	≤0.120	0	0	14	0	0	16
Netilmicin	≤0.120–0.5	≤0.120–0.5	0	0	14	0	0	16
Piperacillin	≤1–4	≤1–4	0	0	14	0	0	16
Piperacillin/tazobactam	≤1–4	≤1	0	0	14	0	0	16
Tigecycline	≤0.060–0.5	0.25–1	0	0	14	2	0	14
Tobramycin	≤0.120–0.25	≤0.120–0.25	0	0	14	0	0	16

S – susceptible; R – resistant; I – intermediate

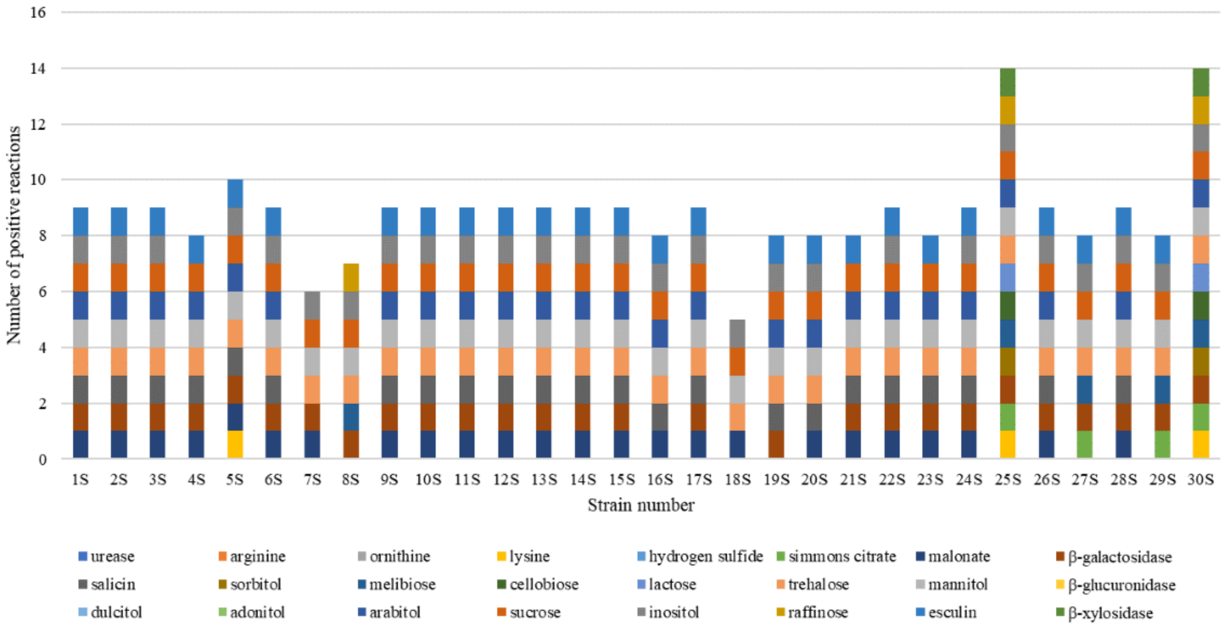

Supplementary Fig. S1. Biochemical features of *Pantoea* strains isolated from food and soil
